# Endurance training significantly increases serum endocan but not osteoprotegerin levels: a prospective observational study

**DOI:** 10.1186/s12872-016-0452-7

**Published:** 2017-01-05

**Authors:** Michael Sponder, Ioana-Alexandra Campean, Michael Emich, Monika Fritzer-Szekeres, Brigitte Litschauer, Jutta Bergler-Klein, Senta Graf, Jeanette Strametz-Juranek

**Affiliations:** 1 Medical University of Vienna, Department of Cardiology, Währinger Gürtel 18-20, 1090 Vienna, Austria; 2 Austrian Federal Ministry of Defence and Sports, Austrian Armed Forces, Brünnerstraße 238, 1210 Vienna, Austria; 3 Medical University of Vienna, Department of Medical-Chemical Laboratory Analysis, Währinger Gürtel 18-20, 1090 Vienna, Austria; 4 Medical University of Vienna, Department of Clinical Pharmacology, Währinger Gürtel 18-20, 1090 Vienna, Austria

**Keywords:** Osteoprotegerin, Endocan, Inflammation, Angiogenesis, Calcification, Physical activity

## Abstract

**Background:**

Endocan (EN) was suggested a potential inflammatory and cardiovascular disease (CVD) marker which might also be involved in renal failure and/or renal failure-associated vascular events. It is not clear whether osteoprotegerin (OPG) is a pro- or anti-atherogenic factor, however, it is agreed upon that OPG is elevated in subjects with increased calcification status. The aim of the study was to investigate the influence of long-term physical activity on serum endocan (EN) and osteoprotegerin-levels.

**Methods:**

One hundred nine subjects were told to increase their amount of physical activity for 8 months by performing 150min/week moderate or 75min/week vigorous exercise. Incremental cycle ergometer tests were performed at the beginning and the end of the study to prove and quantify the performance gain. Blood samples were drawn at baseline and every 2 months for the determination of EN and OPG. To investigate the difference between baseline and 8 months levels of EN and OPG we used a paired sample *t*-test. To investigate the significance of the tendency of the progression (baseline/2 months/4 months/6 months/8 months) we used a Friedman test.

**Results:**

Thirty-eight female and 60 male subjects completed the study. In the group of 61 subjects who had a performance gain by >4,9% EN-levels increased from 146 ± 110 to 196 ± 238 pg/ml (*p* = 0,036) equivalent to an increase of 33,5% but there was no significant change in OPG (4,4 ± 2,4 pmol/l vs. 4,3 ± 2,1 pmol/l; *p* = 0,668).

**Conclusions:**

Physical activity increases significantly EN-levels relativizing the status of EN as proinflammatory factor. EN should rather be considered as a mediator which is involved in several physiological (e.g., angiogenesis) but also pathological processes (e.g., CVD, tumour progression or endothelium-dependent inflammation) and whose expression can be significantly influenced by long term endurance training.

**Trial registration:**

Clinical trial registration number: NCT02097199

Date of trial registration at Clinical Trials.gov: 24.03.2014; last update: 6.1.2016

## Background

OPG, a member of the (TNF)/TNF (tumor necrosis factor) receptor family, is a soluble glycoprotein consisting of 380 amino acids (mature form) which was first characterized by Simonet et al. [1] and Yasuda et al. [2] in 1997/8. OPG is involved in numerous physiological and pathological processes, e.g., atherosclerosis, and physical activity might also have an impact of its circulation amounts. Although it is not clear whether OPG is a pro- or anti-atherogenic factor, it is agreed upon that OPG amounts are elevated in subjects with increased calcification status. The influence of long term physical activity on circulation serum OPG levels remains controversial and finally unclear.

EN, which was previously known as endothelial cell specific molecule-1, ESM-1, is a soluble dermatan sulfate proteoglycan. It is contained in endothelial cells and gets expressed in response to inflammatory cytokines and cell factors such as TNF-alpha, IL (interleukin)1-beta, IL4 or IFN (interferon)-gamma [3]. EN was suggested a potential inflammatory and CVD marker which might also be involved in renal failure and/or renal failure-associated vascular events and the development of cancer and therefore presents one of the most exciting and promising biomarker in present CVD and cancer research.

Physical activity was shown to be one of the most potent preventive strategies against the both “top killers” of humanity: CVD and cancer. According to the current knowledge, sports and physical activity might decrease the risk for coronary heart disease (CHD) by 30–40% [4]. The mechanisms of sports affecting the cardiovascular risk are not completely understood. However, it is known that regular physical activity affects the cardiac cellular and molecular adaption, the vascular adaption [5] and the activity of the immune system. Nowadays, there are several biomarkers available serving as surrogate for physiological and pathological mechanisms in angiogenesis, inflammation and calcification which are all involved in cardiac diseases.

It was therefore the aim of this prospective study to investigate the influence of physical activity on serum EN and OPG levels using a setting with a high number of female and male subjects and a long and close-meshed follow-up.

## Methods

### Description of population

The population has been recruited out of the staff of the Austrian Federal Ministry of Defence and Sports on a first come, first serve basis. The inclusion criteria were: age between 30–65 years, physical ability to perform endurance exercise. Exclusion criteria were: age <30 or >65 years, no ability to perform endurance exercise (due to operations, implantations or any kind of disease that makes endurance training impossible and/or would pose a risk to the participants), current oncological or infectious disease (anamnestic or increased inflammation parameters at baseline). In total 109 subjects were recruited. Eleven subjects did not complete the study for different reasons (accidents, loss of motivation…). Finally, 98 subjects completed the study. However, 37 participants did not achieve a performance gain >4,9% or even had a worse performance compared to the performance at the beginning of the study and were excluded. Therefore, the study population consisted of 19 female and 42 male subjects with at least one classic cardiovascular risk factor defined as follows: overweight (BMI > 25,0 kg/m^2^, hypertension (SBP > 140 mmHg +/− DBP > 85 mmHg at rest/antihypertensive therapy), hyper/dyslipidemia (statin therapy), diabetes mellitus (HbA1_c_ >6,5 rel%/diabetes medication), (ex)smoking (ex-smokers, current smokers), known CVD (anamnestic MI, PCI, CABG, stroke) and positive family history for CVD (MI/CVD/stroke of mother and/or father). Due to the fact that all blood samples were drawn at a not starving state, we did not use data from the lipid profile to diagnose hyper/dyslipidemia but only anamnestic data/medication.

The study was carried out in adherence to the Declaration of Helsinki of the World Medical Association and its later amendments. The protocol has been approved by the Ethical Commission of the Medical University of Vienna and informed consent was obtained from all subjects. The study is registered at Clinical Trails (Clinical trails registration: NCT02097199).

### Measurement of anthropometric data and cycle ergometry

After detailed anamnesis and physical examination including the measurement of height, weight, body water, body muscle mass and body fat (with a diagnostic scale, Beurer BG 16, Beurer GmbH, Ulm, Germany), the subjects had to perform a bicycle stress test (ergometry) at the beginning of the study to define their performance level and to calculate their individual training pulse/target heart rate (using the Karvonen formula with an intensity level of 65–75% for moderate and 76–93% for vigorous intensity). The subjects were let to decide the kind of physical activity/sports, however, they were asked to perform at least 75 min/week of vigorous or 150 min/week of moderate intensity endurance training (or a mixture; strength training was allowed but not mandatory) within the calculated training pulse. A second bicycle stress test was performed at the end of the study (after 8 months) to prove and also quantify exactly and objectively the change/gain in performance. However, the participants also obtained a training diary to record their training effort during the study period. The bicycle stress tests were always ECG-monitored and performed with the same system (Ergometer eBike comfort, GE Medical Systems, Freiburg, Germany) starting with 25 watts and increasing every 2 min by 25 watts (according to the protocol of the Austrian Society of Cardiology which is equal to the guidelines of the European Society of Cardiology). Blood pressure and heart rate were taken every 2 min. Subjects were told to cycle with 50–70 revolutions/min until exhaustion occurred. The target performance was calculated using body surface (calculated according to DuBois formula: body surface (m^2^) = 0,007184 × height [cm] ^0,725^ × weight [kg] ^0,425^) [6], sex and age. An individual target performance of 100% represents the performance of an untrained collective.

### Laboratory analysis

Blood samples were drawn in a not starving state for 5 times: at the beginning of the study (baseline levels) and every 2 months (2 months/4 months/6 months/8 months). All blood samples were taken after 10 min of still lying from an arm vein with a tube/adapter system. Samples for the determination of routine laboratory parameters were analysed immediately after drawing. Samples for the determination of EN (R&D Systems, Minneapolis, USA) and OPG (Biomedica Immunoassays, Vienna, Austria) were centrifuged and frozen immediately after drawing. The analysis was performed according to the manufacturer’s instructions. The EN-assay had a total coefficient of variation (CV) of about 6%; the OPG-assay of 7%.

### Statistical analysis

Statistical analysis was accomplished using SPSS 20.0. Continuous and normally distributed data is described by mean ± standard deviation (SD). Not normally distributed data is described by median/25^th^ quartile/75^th^ quartile. As it was to expect that not all of the subjects would reach an adequate performance gain during the observation period we defined in the forefront a minimum threshold of >4,9% performance gain as significant and excluded participants who did not reach this threshold. To investigate the difference between baseline and 8 month levels of EN and OPG we used a paired sample *t*-test. To investigate the significance of the tendency of the progression (baseline/2 months/4 months/6 months/8 months) we used a Friedman test. All tests were performed in accordance with two-sided testing and *p* values ≤0.05 were considered significant.

## Results

One hundred nine subjects were recruited. Eleven subjects did not complete the study for medical and non-medical reasons. Ninety-eight subjects completed the study but 37 participants did not achieve a performance gain >4,9% or even had a worse performance compared to the performance at the beginning of the study and were excluded. The study population therefore consisted of 61 (19 female and 42 male) subjects with a mean age of 48,9 ± 6,9 years.

Detailed anamnestic and anthropometric data as well as data from the initial laboratory analysis is presented in Table 1. The most prevalent cardiovascular risk factor was overweight (62,3%) followed by positive family history for CVD (42,6%), hypertension (31,1%) and dyslipidemia (27,9%). Data from the ergometer test are shown in Table 2.

**Table 1:** Description of population. Anthropometric data, CVD-risk factors (given as n/% of population), routine laboratory data at baseline and average self reported training effort per month. Data is given as mean +/− standard deviation

*n* (f/m)	61 (19/42)
Risk factors	
Overweight	38/62,3
Hypertension	19/31,1
Dyslipidemia	17/27,9
Hyperglycemia/DM	1/1,6
Current Smoking	11/18
Known CVD	13/21,3
Pos. family history	26/42,6
Anthropometrics	
Age (years)	48,9 ± 6,9
BMI (kg/m^2^)	27,5 ± 4,3
Body water (%)	52,9 ± 5,9
Body fat (%)	29,0 ± 10,4
Body muscle (%)	35,6 ± 4,1
Laboratory analysis	
Erythrocytes (T/l)	4,8 ± 0,4
Haemoglobin (g/dl)	14,2 ± 1,2
Haematocrit (%)	40,6 ± 3,1
Thrombocytes (G/l)	238,6 ± 48,5
Leukocytes (G/l)	6,3 ± 1,3
Na (mmol/l)	141,3 ± 1,6
K (mmol/l)	4,2 ± 0,3
Cl (mmol/l)	101,0 ± 2,0
Ca (mmol/l)	2,3 ± 0,1
Mg (mmol/l)	0,8 ± 0,1
Creatinine (mg/dl)	0,9 ± 0,2
Urea (mg/dl)	16,8 ± 17,9
Uric acid (mg/dl)	5,2 ± 1,2
Albumin (g/l)	45,0 ± 2,3
Lipase (U/l)	40,4 ± 16,8
Cholin esterasis (kU/l)	8,4 ± 1,7
GOT (U/l)	23,9 ± 6,8
GPT (U/l)	26,1 ± 12,0
γ-GT (U/l)	32,4 ± 53,3
Triglycerides (mg/dl)	133,6 ± 73,9
Cholesterol (mg/dl)	201,2 ± 37,2
HDL-cholesterol (mg/dl)	57,1 ± 15,2
LDL-cholesterol (mg/dl)	118,1 ± 34,5
HbA_1_c (rel.%)	5,2 ± 0,3
Average training effort (min./month)	
Moderate intensity	1211 ± 1003
Vigorous intensity	332 ± 359

*GOT* Glutamat-Oxalacetat-Transaminasis, *GPT* Glutamat-Pyruvat-Transaminasis, *γ-GT γ*-Glutamyltransferasis, *HDL-cholesterol* high density lipoprotein cholesterol, *LDL-cholesterol* low density lipoprotein cholesterol

**Table 2:** Results from the bicycle stress tests. Results from the bicycle stress tests at baseline and after 8 months. Data is given as mean +/− standard deviation

	Ergometer test 1	Ergometer test 2	*p*-value
PQ-time rest (ms)	160 ± 23	160 ± 24	0,872
QRS-time rest (ms)	97 ± 13	96 ± 13	0,393
QTc-time rest (ms)	425 ± 19	417 ± 24	0,011
SBP at rest	144 ± 14	133 ± 9,2	<0,001
DPB at rest	86 ± 7	78 ± 7	<0,001
HR at rest	67 ± 7	65 ± 9	0,200
SBP peak	203 ± 20	207 ± 20	0,106
DBP peak	92 ± 12	84 ± 11	<0,001
HR peak	168 ± 16	173 ± 13	0,001
Expected Watts	172 ± 33	169 ± 33	<0,001
Performance (%)	103,5 ± 17,2	117,0 ± 17,6	<0,001

*SBP* systolic blood pressure, *DBP* diastolic blood pressure, *HR* heart rate

Table 3 shows the baseline levels as well as the levels at the different points of follow up (2, 4, 6, 8 months) of EN and OPG. EN-levels increased from 146 ± 110 to 196 ± 238 pg/ml equivalent to an increase of 33,5%. The paired sample *t*-test testing the difference between baseline EN and 8-month-levels showed a significant change (*p* = 0,036). The Friedman test investigating the tendency also showed a significant progression (*p* = 0,013). Concerning OPG, the subjects showed a slight increase from baseline (4,4 ± 2,4 pmol/l) to the 4-month-level (4,6 ± 2,3 pmol/l) and decreased again to 4,3 ± 2,1 pmol/l). Neither the paired sample *t*-test nor Friedman test showed significant changes.

**Table 3:** Progression of EN and OPG levels. EN (pg/ml) and OPG (pmol/l) serum levels at baseline and after 2/4/6/8 months. Data is given as mean ± std.dev. The significance of the difference between baseline levels of OPG and EN were tested using paired sample *t*-test; the significance of the tendency of the progression of baseline levels to levels after 8 months was tested using Friedman test

	Endocan (pg/ml)	Osteoprotegerin (pmol/l)
Baseline	146 ± 110	4,4 ± 2,4
2 months	168 ± 133	4,5 ± 2,7
4 months	168 ± 134	4,6 ± 2,3
6 months	179 ± 224	4,4 ± 2,0
8 months	196 ± 238	4,3 ± 2,1
Paired sample *t*-test	*p*-value = 0,036	*p*-value = 0,668
Friedman test	Chi-Square: 12,696 Asymp. Significance: 0,013	Chi-Square: 8,309 Asymp. Significance: 0,081

## Discussion

We observed a significant difference between the baseline EN levels and the levels after 8 months (+33,5%) in a group of subjects with proven performance gain. Furthermore, the increasing tendency of the increase (measurement every 2 months) was significant. The beneficial effects of exercise on traditional cardiovascular risk factors such as hypertension, diabetes mellitus or dyslipidemia do not fully account for the magnitude of cardiovascular risk reduction. Effects of physical exercise on the vasculature, in particular the vascular endothelium, provide a plausible contribution to the exercise-related risk reduction in cardiac events [7]. The healthy vascular endothelium, as a paracrine and endocrine organ, ensures vascular homeostasis. Any kind of noxa tipping the balance of this sensitive system leads to endothelial dysfunction. Endothelial dysfunction is a well established response to the classic cardiovascular risk factors such as hypertension, diabetes mellitus, dyslipidemia, overweight/adipositas, physical inactivity and smoking and precedes the development of atherosclerosis. It is a basic pathomechanism in cardiovascular disease (CVD) and mainly characterized by increased inflammation, proliferation and coagulation [8]. Several laboratory parameters serve as surrogate for the performance of body systems such as C-reactive protein (CRP) for the inflammation status or high-density and low-density lipoprotein for the lipid profile and play a central role in the pathogenesis, diagnosis but also prevention of CVD.. The preventive effect of physical activity might be caused by several mechanisms affecting inflammation [9], angiogenesis [10] and calcification [11]. Recently published studies suggest an involvement of EN in inflammation, cancer progression an angiogenesis: EN is overexpressed in septic patients, patients with gliomas [12], non-small cell lung cancer tumours [13] and other types of cancer [14] and furthermore was suggested a predictive marker of all-cause mortality and cardiovascular events in patients suffering chronic kidney disease [15]. Several study suggested increased EN levels as common predictor of the endothelium-dependent inflammatory processes. The overexpression of EN might affect two factors which are involved in acute inflammatory response, reperfusion and ischemia injury and antitumor response: LFA-1 (lymphocyte function-associated antigen-1) and ICAM-1 (intercellular adhesion molecule-1). EN may be involved in the regulation of the LFA-1/ICAM-1 pathway, resulting in decreased leukocyte activation by LFA-1, which may affect the lymphocyte homing to sites of inflammation and aggregation. Consequently, leukocyte adhesion and activation may be affected [14]. EN was also shown to correlate with the intima media thickness (an early sign of atherosclerosis) and BMI in patients suffering systemic lupus erythematosus [16]. These findings consolidated the position of EN as atherosclerosis surrogate. Interestingly, it was also shown that high levels of EN expression correlate with angiogenic factors, such as VEGF-A (vascular endothelial growth factor A) and FGF-2 (fibroblast growth factor-2) and might therefore have an influence on angiogenesis, however, the exact mechanism is not known yet [13]. A recently published study by Cimen et al. [17] showed that patients with ischemic heart disease and patients with microvascular angina have significantly higher plasma EN levels compared to controls. They furthermore found an independent positive correlation between EN levels and the SYNTAX score.

Taken together the results of the mentioned studies, EN is somehow “tainted with a bad repute”. However, as mentioned above, EN also shows a potential anti-inflammatory activity through inhibition of the LFA-1–dependent leukocyte function [3] and might collude with VEGF leading to increased angiogenesis. Since physical activity is well known to counteract/retard atherosclerotic processes, improve angiogenesis and vascularization and acts preventive against several types of cancer, our results would be contradictory to the current knowledge. Our results show a physical activity induced increase in serum EN levels by about 33,5%. The EN increase might represent exercise-derived activation of the vascular endothelium. We believe that EN plays also an important role in physiological processes and that a certain amount of circulation EN is absolutely necessary e.g., for adequate angiogenesis, anti-inflammation and probably wound healing. Microarray analysis has revealed that during angiogenesis tip cells specifically express endocan in much high level in comparison to rest of the vasculature [18].

EN should rather be considered as a mediator which is involved in several physiological and pathological processes and whose expression can be significantly influenced by long term physical activity.

However, within our study population, we could not state an influence of physical activity on circulating OPG amounts although the study population was quite large, the follow up close-meshed and the observation period long. As vascular calcification is a process with slow progression it is conceivable that the observation period was too short to state an impact of sports on OPG levels.

## Conclusion

In conclusion, the influence of sports on EN seems to appear quite promptly (within 2 months). These results relativize the “bad repute” as pro-inflammatory and cancer-promoting factor. EN could rather be considered as a mediator which is involved in several physiological (e.g., angiogenesis) but also pathological processes (e.g., tumour progression or endothelium-dependent inflammation) and whose expression can be significantly influenced by long term endurance training. There is more data needed concerning the molecular mechanisms in which EN is involved. Concerning OPG, we could not observe a physical activity induced increase or decrease of OPG serum levels.

### Limitations

The present study has several limitations: First, although the original study population was quite large for a prospective study, only 61 subjects improved their performance by >4,9%. Second, as vascular calcification is a process with slow progression, it is conceivable that the observation period was too short. Third, several other factors that were not controlled might have an influence on circulating serum OPG and EN levels.

## Abbreviations

CHD: Coronary heart disease
CRP: C-reactive protein
CVD: Cardiovascular disease
ELISA: Enzyme-linked immunosorbent assay
EN: Endocan
ESM-1: Endothelial specific molecule-1
FGF: Fibroblast growth factor
ICAM: Intercellular adhesion molecule
IFN-gamma: Interferon gamma
IGFR: Insulin-like growth factor receptor
IL: interleukin
LFA-1: Lymphocyte function-associated antigen-1)
OPG: Osteoprotegerin
RANKL: Receptor activator of nuclear factor kappa-B ligand
SD: Standard deviation
TNF: Tumor necrosis factor
TRAIL: TNF-related apoptosis inducing ligand
VEGF: Vascular endothelial growth factor
